# Panel data on the impact of democracy and political stability on economic growth in the MENA region over the years 1983–2022

**DOI:** 10.1016/j.dib.2023.109389

**Published:** 2023-07-07

**Authors:** Brahim Zirari, Youcef Souar, Roucham Benziane, Abdelhak Lefilef

**Affiliations:** a Faculty of Economics, Dr Tahar Moulay University, Algeria; b Faculty of Economics and Management, University of Bechar, Algeria; c Abdelhafid Boussouf University Center, Mila, Algeria

**Keywords:** Economic Growth, Democracy, Political Stability, Secondary Data, Panel Data

## Abstract

This panel dataset contains information collected from five distinct sources on the impact of democracy and political stability on economic growth in 15 emerging countries in the MENA region from 1983 to 2022. The dataset includes information about GDP per capita, inflation, trade, population growth, government expenditure, participatory democracy, deliberative democracy, egalitarian democracy, institutionalized autocracy, and political stability. Data analysis was carried out utilizing R statistical software to ensure the accuracy of the data by imputing missing data using the monotone REG approach, allowing future researchers to investigate the linkage between political factors and growth in various scenarios. Data are provided in two sheets, one containing the data before the missing value imputation and another after the monotone REG imputation. This dataset is a companion for an unpublished thesis titled “Democracy, Political Stability, and Economic Growth.”

**Specifications Table**

**Table:** 

Subject	Economics and Econometrics
Specific subject area	Economic Analysis, Political Economy
Type of data	Table, xlsx files Figures
How the data were acquired	Statistics were acquired from the following sources: the World Bank Development Indicators (WDI)(1), the World Bank Governance Indicators (WGI)(2), the Penn World Table (PWT)(3), Polity5 from The Integrated Network for Societal Conflict Research (INSCR)(4), and the Varieties of Democracy (V-Dem)(5). Some data analysis was performed using R Software.
Data format	Filtered
Description of data collection	The provided sheets include information about GDP per capita (GROWTH), Inflation (INF), Trade (TRADE), final Government consumption (GOVTSIZE), Population growth (POP), Democracy (DEM), Political stability (PST), and Institutionalized Autocracy (AUTOC). These data were collected from various sources for 15 MENA countries between 1983 and 2022. Data analysis has shown some missing data for some variables; hence, the “mice package” using R software was adopted to impute missing data. The data are presented in two versions, before and after the imputation.
Data source location	*15 MENA countries: Algeria, Tunisia, Morocco, Egypt, Lebanon, Sudan, Syria, Iraq, Iran, Bahrain, Jordan, Kuwait, Oman, Qatar, and Saudi Arabia.* Official data sources: (1) https://databank.worldbank.org/source/world-development-indicators (2) https://databank.worldbank.org/source/worldwide-governance-indicators (3) https://www.rug.nl/ggdc/productivity/pwt/ (4) https://www.systemicpeace.org/ (5) https://v-dem.net/
Data accessibility	Repository name: “Panel_democ_stability_growth_MENA_Over_1983_2022”, Mendeley Data Data identification number: 10.17632/vhh9cg2wzt.3 Direct URL to data: https://data.mendeley.com/datasets/vhh9cg2wzt/3

## Value of the Data

• These data are helpful mainly for understanding the impact of political factors, particularly democracy and political stability, on economic growth.
• Researchers and policymakers can benefit from these data.
• The data can be used to validate and extend existing theories on the impact of political factors on GDP per capita growth, potentially leading to the development of effective economic policies and, hence, long-term development.
• The data can be reanalyzed using different analytical methods or combined with other datasets to explore new research questions and hypotheses.
• Future experiments can be designed based on these data to explore the causal nexus between democracy-economic growth.

## 1 Objective

The primary objective behind generating this dataset is to explore the impact of the participatory democracy index, egalitarian democracy index, deliberative democracy index, political stability index, and institutionalized autocracy on GDP per capita in 15 MENA countries between 1983 to 2022. This data article adds value to the scientific community by offering a rich and comprehensive dataset to analyze further and use to validate existing theories or develop new hypotheses regarding the linkage between political factors and economic growth. The dataset can also serve as a foundation for future research, enabling the design of experiments to investigate causal relationships or the impact of democracy and economic growth.

## 2 Data Description

The dataset incorporates ten variables: GDP per capita, trade, inflation, population growth, government final consumption expenditure, deliberative democracy index, participatory democracy index, egalitarian democracy index, political stability, and institutionalized autocracy index. The economic variables suggested by [1] were obtained from the World Bank Development Indicators (WDI) and the Penn World Table (PWT). For political stability, data were collected from the World Bank Government Indicators (WGI) following [2]. The main variable in our analysis is democracy, as previous literature shows conflicting views on the effect of democracy on growth. Therefore, information for democracy was collected from V-Dem, the most relevant metric for democracy, as stated in [3]—Finally, Institutionalized autocracy was assembled from Polity5. The explanations and sources of the variables are provided in Table 1.

**Table 1:** Description of the variables in (Panel_democ_stability_growth_MENA_Over_1983_2022).

Variable	Label	Definition	Source
Economic growth	GROWTH	GDP per capita annual growth.	WDI and PWT
Inflation	INF	The annual change in consumer price index (CPI)	WDI
Trade	TRADE	The total of goods and services imported and exported (divided by GDP)	WDI
Government final consumption to GDP	GOVTSIZE	The revenue reflects government spending on goods and services.	WDI
Population Growth	POP	The annual total growth of the population.	WDI
Political stability	PST	Values range between -2.5 and +2.5, with -2.5 being the most unstable situation and +2.5 indicating the most stable.	WGI
Participatory democracy index	PARTIPDEM	Citizens' engagement in electoral and non-electoral processes.	V-DEM
Deliberative democracy index	DELIBDEM	The democratic deliberative principle concerns how decisions are made in society.	V-DEM
Egalitarian democracy index	EGALDEM	The degree to which social groups have equal access to resources, authority, and guaranteed rights and freedoms.	V-DEM
Institutionalized Autocracy	AUTOC	The restricted competitive political participation and regularized the chief executive selection process.	Polity5

## 3 Experimental Design, Materials and Methods

**Fig. 1:**
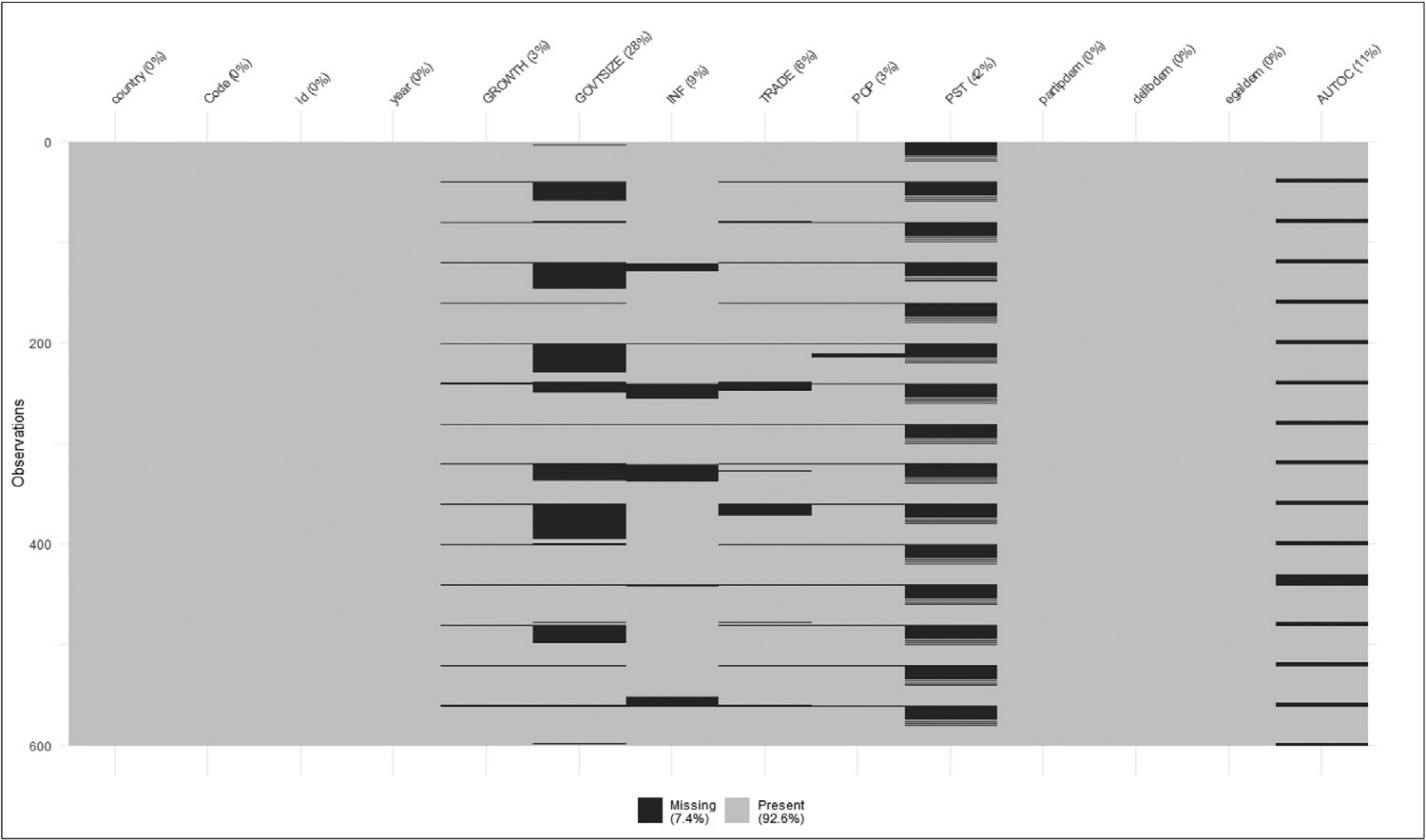
Percentage of missing values for each variable.

Data collection for this study was conducted from various sources. The study sample represents 15 MENA countries over 39 years, mainly from 1983 to 2022, with 600 observations. Since the existence of missing data in political-economic datasets might challenge the empirical analysis of quantitative research, resulting in incorrect parameter estimations, information loss, higher standard errors, and decreased generalizability [4], a diagnostic analysis was applied using R software to analyze the collected data and assess the suitability of variables for the research. Fig. 1 depicts the percentages of missing values, with 92.6% being present values and 7.4% being missing, demonstrating that imputation is essential to replace missing data and enable a rigorous study.

**Fig. 2:**
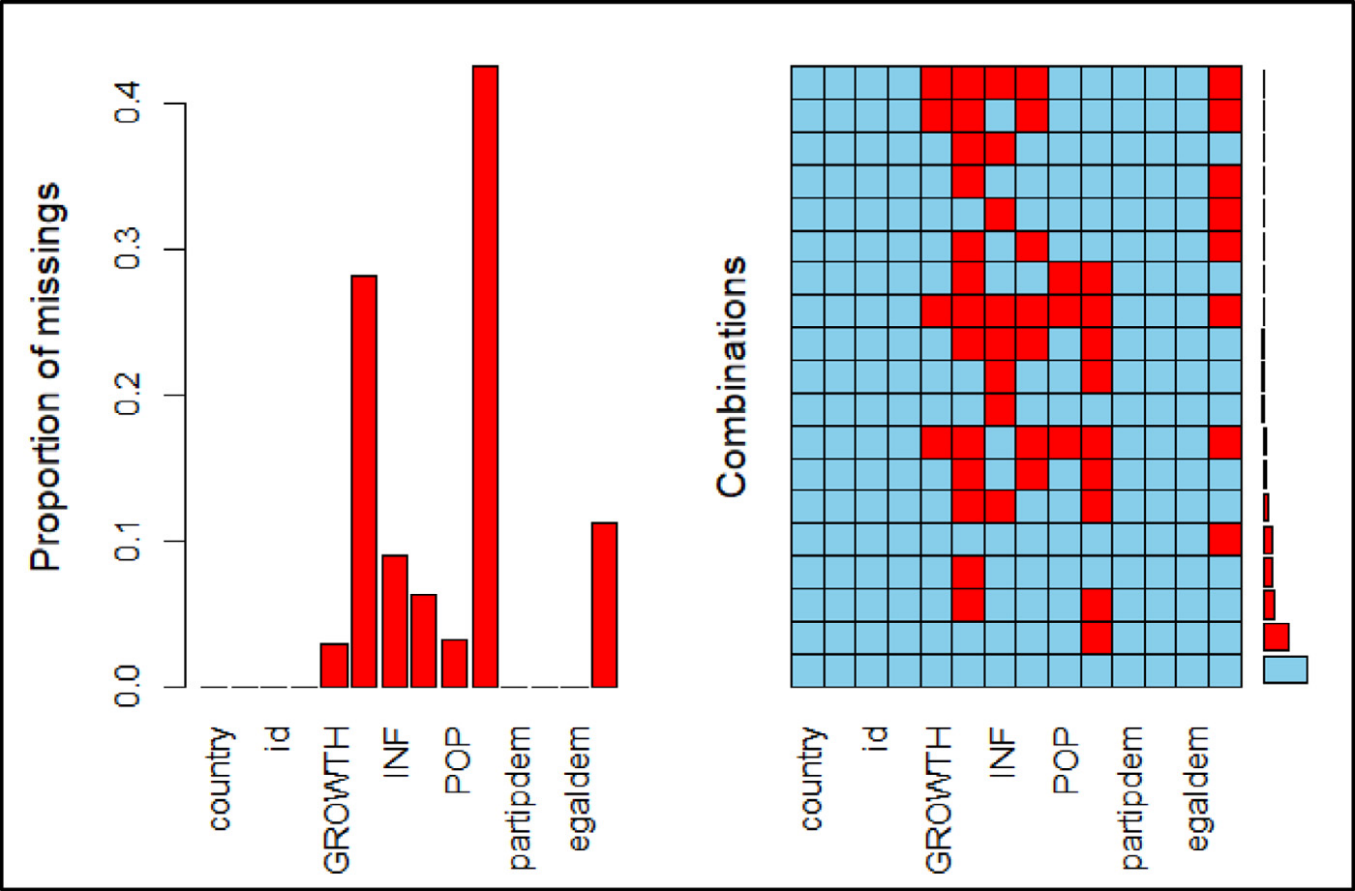
Missing values pattern.

Fig. 2 shows the missing data distribution pattern, which helps researchers determine the types of missing data and, as a result, the most appropriate imputation strategy. Missing data is classified into monotone and non-monotone patterns, and each pattern includes suggested imputation procedures for both.

**Fig. 3:**
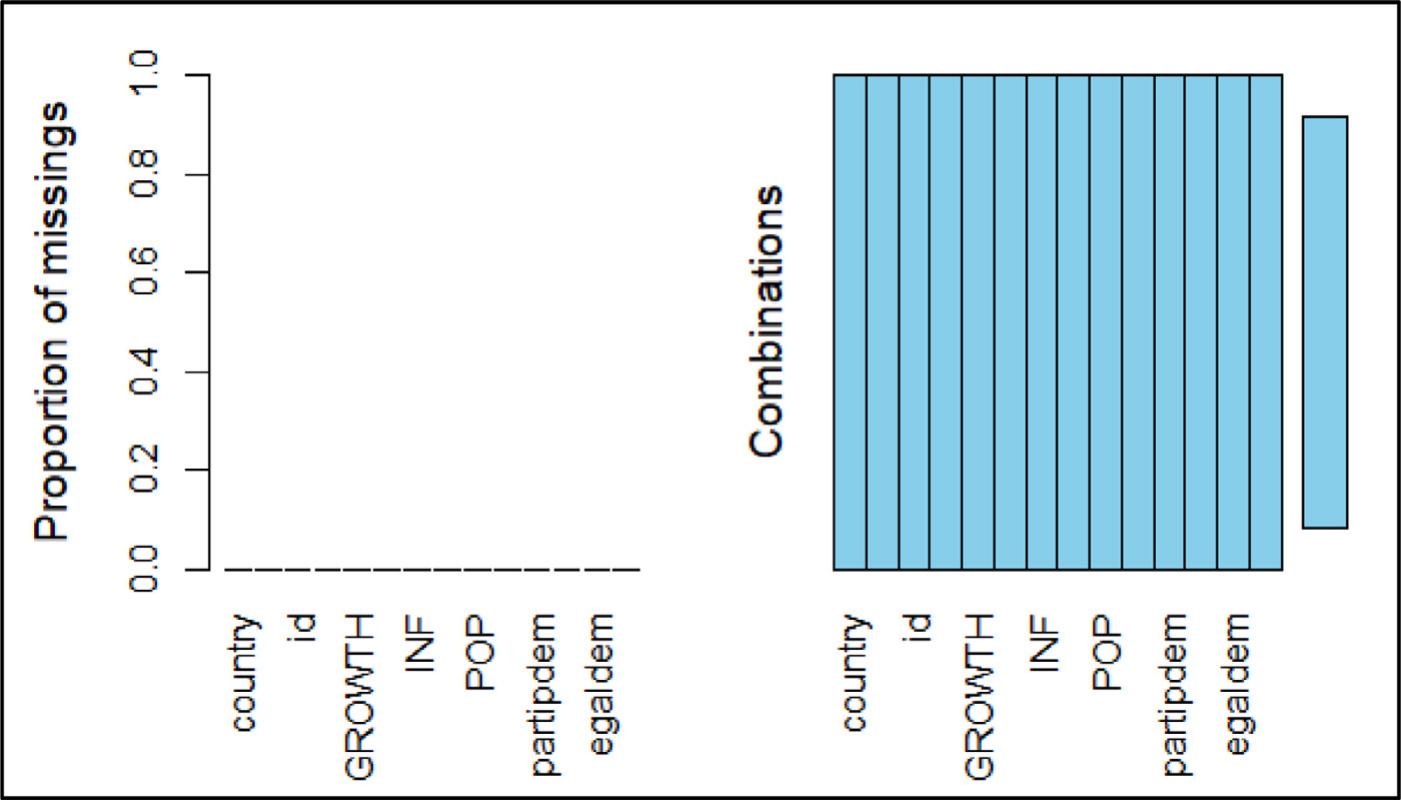
Missing data pattern using monotone REG imputation method.



The collected data show a monotone trend; hence, the “mice” package using R software was adopted to impute missing data using a regression method called “monotone REG” through the following code (results of the monotone REG imputation are displayed in Fig. 3).

**Fig. 4:**
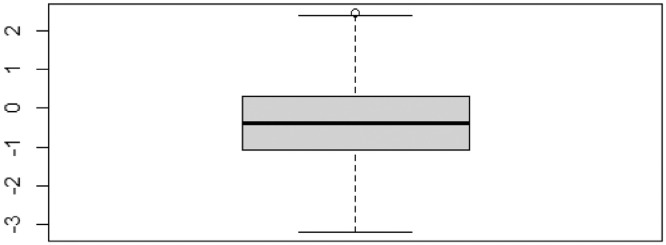
Outliers analysis for PST.

As a final step, outliers analysis was performed the political stability index (PST) (see Fig. 4), before running the empirical analysis due to its significant imputation values compared to the rest of the variables. The outliers analysis along other diagnostics should be performed for all imputed variables as we used only one data imputation from the fifth suggestions (m=5). In this regard, researchers can use the original dataset (panel_democ_before_imp) to control for results, avoid misleading data, and hence ensure robust data for the empirical investigations.

To summarize, this data paper provides two XLS datasets. The first (panel_democ_before_imp) is a panel data that give information before missing value imputation. The second dataset (panel_democ_after_imp) contains the data after adopting the “mice package” as a missing value imputation method.

## Ethics Statements

The authors were carried out in conformity with the Declaration of Helsinki.The authors have read and follow the ethical requirements for publication in Data in Brief and confirming that the current work does not involve human subjects, animal experiments, or any data collected from social media platforms.

## Cite this Dataset

B. Zirari, Y. Souar, R. Benziane, A. Lefilef, Panel_democ_stability_growth_MENA_Over_1983_2022, Mendeley Data, V3, 2023. https://doi.org/10.17632/vhh9cg2wzt.3.

## CRediT authorship contribution statement

**Brahim Zirari:** Software, Validation, Formal analysis, Resources, Data curation, Writing – original draft, Writing – review & editing. **Youcef Souar:** Conceptualization, Methodology, Visualization, Supervision. **Roucham Benziane:** Conceptualization, Methodology, Validation, Formal analysis, Investigation, Writing – original draft, Writing – review & editing, Visualization, Supervision, Project administration. **Abdelhak Lefilef:** Methodology, Investigation, Writing – original draft, Writing – review & editing.

## Declaration of Competing Interest

The authors declare that they have no known competing financial interests or personal relationships that could have appeared to influence the work reported in this paper.

## Data Availability

Panel_democ_stability_growth_MENA_Over_1983_2022 (Original data) (Mendeley Data).
